# Association between COVID-19 emergency declarations and physical activity among community-dwelling older adults enrolled in a physical activity measurement program: Evidence from a retrospective observational study using the regression discontinuity design

**DOI:** 10.1186/s12889-023-15932-0

**Published:** 2023-05-30

**Authors:** Ippei Chiba, Masayoshi Takahashi, Sangyoon Lee, Seongryu Bae, Keitaro Makino, Osamu Katayama, Kenji Harada, Kouki Tomida, Masanori Morikawa, Yukari Yamashiro, Naoto Takayanagi, Motoki Sudo, Hiroyuki Shimada

**Affiliations:** 1 Department of Preventive Gerontology, Centre for Gerontology and Social Science, National Centre for Geriatrics and Gerontology, 7-430, Morioka-Cho, Obu City, Aichi 474-8511 Japan; 2 Department of Preventive Medicine and Epidemiology, Tohoku Medical Megabank Organization, Tohoku University, Sendai, 980-8573 Japan; 3 School of Information and Data Sciences, Nagasaki University, 1-14 Bunkyo, Nagasaki City, , 852-8521 Japan; 4 Department of Health Care and Science, Dong-A University, Nakdong Dae-Ro 550-37, Saha-Gu, Busan, 49315 Korea; 5 Japan Society for the Promotion of Science, Chiyoda-Ku, Tokyo, Japan; 6 Tokyo Research Laboratories, Kao Corporation, Sumida-Ku, Tokyo, 131-8501 Japan

**Keywords:** Physical activity, COVID-19, Emergency declaration, Older adults, Regression discontinuity design, Multiple imputation

## Abstract

**Background:**

The current study examines the negative impact of the coronavirus disease 2019 (COVID-19) emergency declarations on physical activity among the community-dwelling older adults, the participants of a physical activity measurement program, in Japan.

**Methods:**

This retrospective observational study included 1,773 community-dwelling older adults (aged 74.6 ± 6.3 years, 53.9% women) who had participated in the physical activity measurement project from February 2020 to July 2021. We measured physical activity using a tri-axial accelerometer during 547 consecutive days. Three emergency declarations, requesting people to avoid going outside, occurred during the observational period. We multiply-imputed missing values for daily physical activity, such as steps, light physical activity (LPA), and moderate-to-vigorous physical activity (MVPA) for several patterns of datasets according to the maximum missing rates on a person level. We mainly report the results based on less than 50% of the maximum missing rate (*n* = 1,056). Other results are reported in the supplemental file. Changes in physical activity before and after the start of each emergency declaration were examined by the regression discontinuity design (RDD) within 14-, 28-, and 56-day bandwidths.

**Results:**

For all the participants in the multiply-imputed data with the 14-day bandwidth, steps (coefficients [${\widehat{\gamma }}_{1}$]$,$ 964.3 steps), LPA (${\widehat{\gamma }}_{1},$ 5.5 min), and MVPA (${\widehat{\gamma }}_{1},$ 4.9 min) increased after the first emergency declaration. However, the effects were attenuated as the RDD bandwidths were widened. No consistent negative impact was observed after the second and third declarations. After the second declaration, steps (${\widehat{\gamma }}_{1},$-609.7 steps), LPA (${\widehat{\gamma }}_{1},$-4.6 min), and MVPA (${\widehat{\gamma }}_{1},$-2.8 min) decreased with the 14-day bandwidth. On the other hand, steps (${\widehat{\gamma }}_{1},$ 143.8 steps) and MVPA (${\widehat{\gamma }}_{1},$ 1.3 min) increased with the 56-day bandwidth. For the third declaration, LPA consistently decreased with all the bandwidths (${\widehat{\gamma }}_{1},$-2.1, -3.0, -0.8 min for the 14, 28, 56-day bandwidth), whereas steps (${\widehat{\gamma }}_{1},$-529 steps) and MVPA (${\widehat{\gamma }}_{1},$-2.6 min) decreased only with the 28-day bandwidth.

**Conclusions:**

For the community-dwelling older adults who regularly self-monitor their physical activity, the current study concludes that there is no evidence of consistently negative impacts of the emergency declarations by the COVID-19 pandemic.

**Supplementary Information:**

The online version contains supplementary material available at 10.1186/s12889-023-15932-0.

## Introduction

The coronavirus disease 2019 (COVID-19) pandemic began in 2020, and the number of infected patients reached 290 million by December 2021 [1]. In Japan, the first COVID-19 patient was identified in January 2020 with subsequent spread in the first half of 2020 [2]. To prevent the spread of the pandemic, people were forced to isolate and maintain a certain distance from each other. The COVID-19 pandemic altered the daily living, social connection, and mental conditions of older adults [3–7]. Therefore, it is important to attenuate the adverse effect of the COVID-19 pandemic on older adults.

Several countries implemented a city lockdown to avoid contact among people. For example, in China and in the UK, physical activity dramatically decreased during the city lockdown period [8, 9]. In Japan, the government announced an emergency declaration under which people were requested to refrain from going outside and to avoid “crowded places,” “closed spaces,” and “close-contact with other people” (known as the 3Cs). The emergency declaration in Japan was unique because it was not mandatory; nevertheless, the infection rate of COVID-19 was estimated to decrease after the emergency declaration [10]. As reported, compared with the period before COVID-19 in Japan, the total physical activity among older adults decreased during the emergency declaration, and some individuals did not regain the same amount of physical activity even after the declaration ended [11]. Physical inactivity is a significant risk factor of disability for non-communicable diseases, such as cardiovascular disease, chronic metabolic diseases, and mental dysfunction [12]. In addition, physical inactivity leads to disability in older adults via progression of the frail cycle, including the loss of muscle mass, strength, and function [13]. Therefore, there is a possibility that the number of older adults with progressive disease and disability may increase due to the physical inactivity during and after the emergency declarations.

Although previous studies investigated the effect of emergency declarations and city lockdowns (to prevent the spread of COVID-19) on physical activity, the following four important issues remain unsolved.

First, few studies have used objective measures such as an accelerometer. Most studies that reported the reduction in physical activity after the emergency declarations or city lockdowns investigated physical activity using a questionnaire via an online survey or phone interviews [11, 14, 15]. The current study is important in that physical activity intensity was objectively measured by an accelerometer.

Second, little is known about the adverse effect of the COVID-19 emergency declarations on the different levels of physical activity intensity, despite the fact that the effects of physical activity differ depending on its intensity. The level of daily life activity is generally of light intensity, while the level of sport or exercise activity is of moderate-to-vigorous intensity [16]. Generally, physical activity of moderate-to-vigorous intensity is recommended for older adults. The physical activity guideline by the World Health Organization recommended moderate-to-vigorous intensity for older adults because it reduces mortality, risk of several diseases, and physical dysfunction [17]. Furthermore, light intensity physical activity (LPA), generally identified as 2.9 metabolic equivalents of task (METs) or below, reduces the risk of cardiometabolic risk factors, mental health, and disability for older adults [18, 19]. The current study is important in that different levels of physical activity intensity were measured by an accelerometer.

Third, previous studies in Japan were unsuccessful in claiming causality because they were based on a simple one-group, pre-post design [11, 20]. In order to identify the effect of the emergency declarations, causality should be identified using objective, consecutive, and longitudinal intensity-specific physical activity measurements. The current study is important in that causal effects of the emergency declarations were investigated by the regression discontinuity design (RDD), a quasi-experimental design.

Fourth, the emergency declarations in Japan were not mandatory in requiring the people to stay at home, compared with the mandatory city lockdown in other countries. Therefore, the Japan emergency declarations are expected to have an effect on physical activity that is different from those in other countries.

Since 2011, we have conducted the cohort study for community-dwelling older adults, titled National Center for Geriatrics and Gerontology Study of Geriatric Syndrome (NCGG-SGS) [21]. From 2015 to 2021, a sub-cohort, which involved a longitudinal physical activity measurement program through tri-axial accelerometers, was initiated in the rural city. The COVID-19 pandemic in Japan began to spread in February 2020, allowing for detailed physical activity data to be gathered. Hence, we can examine these unresolved issues, using the cohort data from February 2020 to July 2021.

We hypothesized that the emergency declarations in Japan would have a negative impact on physical activity. The current study contributes to the promotion of physical activity for older adults by an estimation of the adverse effect of the emergency declarations under the COVID-19 pandemic on physical activity. This study examined the effect of the emergency declarations on the different levels of physical activity intensity, measured by an accelerometer, using a quasi-experimental design in community-dwelling older adults who participated in the physical activity measurement program.

## Material and methods

### Participants

This retrospective observational study included community-dwelling older adults aged ≥ 60 years living in Takahama City, Japan, who were enrolled in a sub-cohort of the NCGG-SGS [21]. Baseline surveys, including 4,092 older adults, were performed from September 2015 to February 2017 at community centers. This cohort was involved with a longitudinal physical activity measurement program, which asked the participants to wear the accelerometers to longitudinally investigate their daily physical activity, after the baseline survey until March 2021. Also, the participants were given a monthly report of their physical activity to monitor their own physical activity levels via an exclusive reading device at community facilities, such as public spaces, drug stores, cafe, and grocery stores. The monthly report of the physical activity included daily steps, daily gait speed (measured by the accelerometer), and target numbers of daily steps and gait speed for the current and following month. Finally, 1,773 participants had wearing time detected valid physical activity data that were observed for at least one day, from February 2020 to July 2021.

As noted above, the baseline survey originally included 4,092 participants. We recorded physical activity from February 2020 to July 2021, which is 547 days. Therefore, the ideal sample size is 4,092*547 = 2,238,324 person-days. However, only 1,773 participants had recorded at least one day of physical activity, which makes our sample size 1,773*547 = 969,831 person-days, after multiple imputation. This means that 2,319 participants had no data on physical activity, which makes it impossible to use multiple imputation to take care of the missing values of physical activity for these 2,319 participants. When there are zero observations, it is impossible to create an imputation model.

Among these 1,773 participants, there were 30 participants whose physical activity was recorded only one day in 547 days. For these participants, it is still possible to use multiple imputation to take care of the missing values, but 546 observations for each of the 30 participants are simulated values after multiple imputation. This means that the maximum missing rate at the person level is 99.82%. This may pose a serious problem in inference. There are no ideal solutions to this problem, and we must face trade-offs. Therefore, we decided to conduct several analyses based on the following four patterns of data.

First, there are 1,773 participants whose physical activity was recorded at least 1 day in 547 days. This means that the maximum missing rate at the person level is about 99.82%. Our sample size after multiple imputation is 1,773*547 = 969,831 person-days. We call this Case 1 in the Supplemental file.

Second, there are 1,289 participants whose physical activity was recorded at least 137 days in 547 days. This means that the maximum missing rate at the person level is about 75%. Our sample size after multiple imputation is 1,289*547 = 705,083 person-days. We call this Case 2 in the Supplemental file.

Third, there are 1,056 participants whose physical activity was recorded at least 274 days in 547 days. This means that the maximum missing rate at the person level is about 50%. Our sample size after multiple imputation is 1,056*547 = 577,632 person-days. We call this Case 3, and we decided to choose Case 3 as our main analysis to be reported in the main body of this article, because several short-term measurement studies used 50% or more adherence data, despite non-imputed data. [22]

Fourth, there are 768 participants whose physical activity was recorded at least 411 days in 547 days. This means that the maximum missing rate at the person level is about 25%. Our sample size after multiple imputation is 768*547 = 420,096 person-days. We call this Case 4 in the Supplemental file.

This study was approved by the appropriate institutional review board (approval number: 1440–2). The opt-out approach for informed consent was waived and approved by the institutional review board of National Center for Geriatrics and Gerontology due to retrospective nature of study.

### Physical activity measurement

This study measured the amount of time engaged in physical activity using a tri-axial accelerometer (HW-100: Kao Corporation, Tokyo, Japan), which was set at a four-second epoch length. The intensity level of an activity was calculated according to the same algorithm implemented in the Kenz Lifecoder (Suzuken Corporation, Limited: Aichi, Japan), on a scale of 0.5 to 9.0, where 0.5 and 9.0 are the minimal and maximal intensity of movement, respectively [23].

In the current study, LPA was defined as intensity levels of 1.0 to 3.0 (corresponds to ≥ 1.6 to ≤ 2.9 METs), while moderate-to-vigorous physical activity (MVPA) was 4.0 to 9.0 (corresponds to ≥ 3.0 METs). We calculated the daily time taken to perform LPAs and MVPAs by adding up all epochs in a day according to each intensity. At the end of the baseline survey, we asked the participants to wear the accelerometers on their waists throughout the day, except at bath time. The daily accelerometer data were considered valid if the participants wore the device 10 h or more per day [22]. Subsequently, we calculated the daily time engaged in LPA and MVPA.

### Timeline

**Fig. 1:**
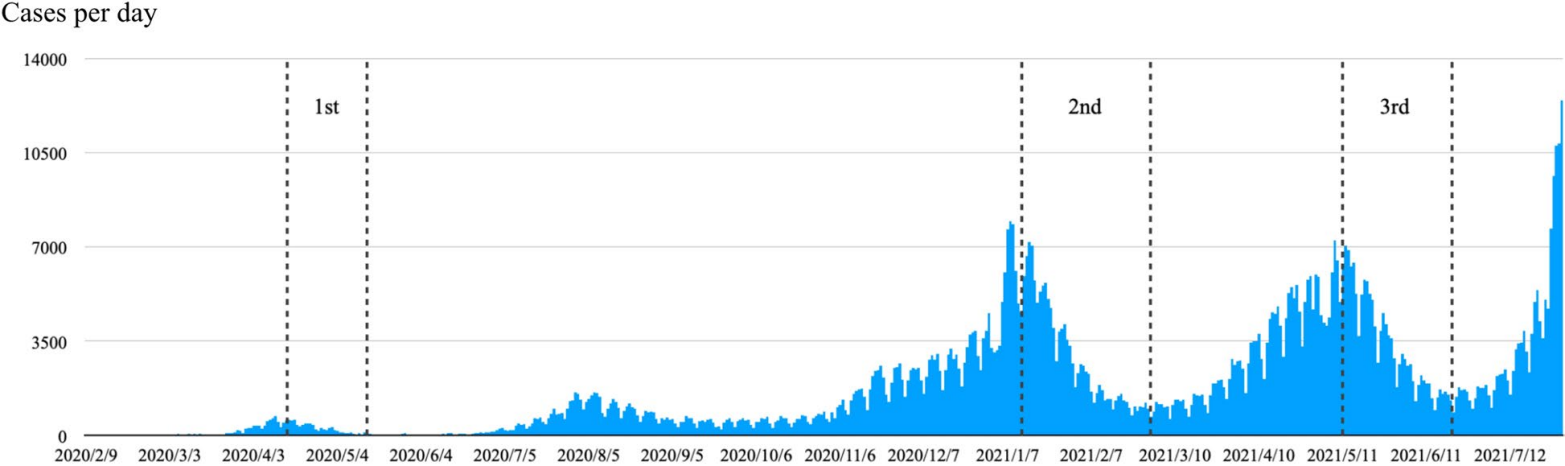
Number of newly diagnosed COVID-19 cases per day in Japan

Takahama City, a regional city in Aichi Prefecture, Japan, has a population of about 49,000 and an area of 13 ${\mathrm{km}}^{2}$. Cases of COVID-19 infection recorded during this study period (from February 2020 to July 2021) were: 261 for Takahama City; 54,093 for Aichi Prefecture (with a population of approximately 7,500,000); and 1,733,536 for Japan (with a population of approximately 125,800,000) [2, 24]. The Japanese government announced the emergency declarations three times during the observation period in at least one region as follows: (1) from April 16 to May 21, 2020; (2) January 13 to February 28, 2021; and (3) May 12 to June 20, 2021. The number of infected people during the three emergency declarations is graphically presented in Fig. 1. Public facilities and spaces were closed, and residents were asked to voluntarily avoid going outside during the emergency declarations, although there was no legal restriction. During the emergency declarations, some facilities with the accelerometer reading device were also closed; thus, the participants had to read the accelerometer at the open facilities, such as drug and grocery stores.

### Statistical analysis

The participants’ baseline NCGG-SGS characteristics that were less likely to fluctuate over time included age, sex, and educational levels. Data in Table 1 are described as mean (SD: standard deviation) for continuous variables and numbers (%) for categorical variables. There were some missing values in education, steps, LPA, and MVPA; therefore, we multiply-imputed the missing data using multiple imputation (MI) based on the expectation–maximization with bootstrapping (EMB) algorithm [25]. This is a fast and reliable MI algorithm, especially for time-series, cross-sectional data [26, 27]. The number of multiple imputation was set to 100, i.e., M = 100 [28]. Since zero is a logical lower bound for age, steps, LPA, and MVPA, we used this prior information to draw imputations from a truncated distribution. Our imputation model also included second and third order polynomials so that the variations across time were accounted for [25, 28]. The mean number of valid physical activity measurement days per participants was 313.4 with the standard deviation of 194 days (ranged 1 to 547 days). Mean wearing time was 839.5 with the standard deviation of 155.2 min per day of valid measurement day. Due to unclear appropriate adherence for prolonged and consecutive physical activity measurement, we generated several multiply-imputed datasets based on the maximum missing rates on a person level during the observational period. Our analyses were conducted on four imputed datasets: those with the maximum missing rates of less than 99.82% (at least 1 day: Case 1), 75% (at least 137 days: Case 2), 50% (at least 274 days: Case 3), and 25% (at least 411 days: Case 4). We mainly report the results based on less than 50% of the maximum missing rate (*n* = 1,056: Case 3), because several studies used the 50% or more valid measurement observations during observational period as valid data, despite short-term measurement and non-imputed data [22]. Other results are reported in the Supplemental file.

**Table 1:** Participants’ characteristics (*n* = 1,056) for Case 3

Variables	mean	SD or %	missing rate	mean^**^	SD or %^**^
Age, years	74.6	(5.9)	0.0		
Age ≥ 75, n (%)^*^	503	(47.6)	0.0		
Women, n (%)^*^	597	(56.5)	0.0		
Education, years	11.5	(2.4)	0.3	11.5	(2.4)
Step counts at first 14 days, steps/day					
All participants	6552.2	(4130.0)	16.1	6698.1	(4043.6)
Men	7488.2	(4631.3)	17.3	7596.2	(4467.6)
Women	5850.7	(3551.9)	15.2	6007.6	(3532.7)
LPA time at first 14 days, min/day					
All participants	42.8	(21.9)	16.1	43.2	(21.6)
Men	43.3	(24.2)	17.3	43.6	(23.6)
Women	42.5	(20.0)	15.2	42.9	(20.0)
MVPA time at first 14 days, min/day					
All participants	33.6	(27.7)	16.1	34.5	(27.1)
Men	39.7	(31.9)	17.3	40.4	(30.7)
Women	29.0	(23.1)	15.2	30.0	(23.1)

SD, standard deviation; LPA, light intensity physical activity; MVPA, moderate-to-vigorous intensity physical activity

Education data were collected during the baseline survey conducted from 2015 to 2017

Age information was collected as of February 1st, 2022, while physical activity data were gathered between February 1st and 14th of the same year

Mean number of valid measurement days: 458.9 ± 79.8 days, and mean wearing time was 851.2 with standard deviation of 154.4 min per day of valid measurement day

^*^Data for "Women, yes" and "Aged 75 year or above" are presented as n (%)

^**^Multiply-imputed data (M = 100)

**Fig. 2:**
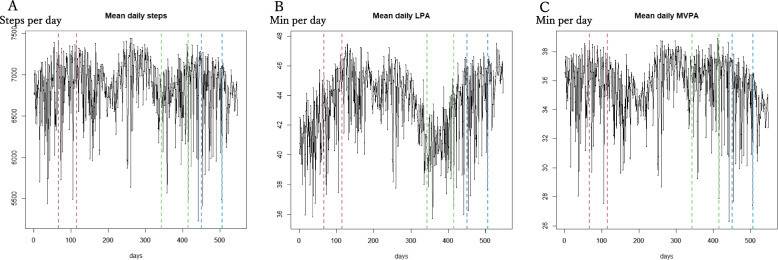
Mean daily physical activity of participants for all periods. **A** daily steps; **B** daily time engaged in light intensity physical activity (LPA); **C** daily time engaged in moderate-to-vigorous intensity physical activity (MVPA)

At descriptive analysis, mean daily steps, mean daily time engaged in LPA, and mean daily time engaged in MVPA were plotted during the measurement period (Fig. 2), to display the first 100 multiply-imputed data only. In other words, we created 100 graphs similar to Fig. 2.

The effect of the emergency declarations was examined by the sharp RDD using the declaration start date as the cutoff point. RDD is known to be one of the strongest quasi-experimental designs, because it utilizes local randomization around the cutoff point [29, 30]. The current study is based on observational data, where we observed physical activity among community-dwelling older adults from February 2020 to July 2021. During these 547 days, there were three interventions, i.e., the emergency declarations, which created three cutoff points. This allows us to use the sharp RDD to estimate the causal effect of the emergency declarations on physical activity among community-dwelling older adults, by comparing the change in physical activity right before and right after the emergency declarations. By the virtue of RDD, local randomization is expected to be achieved, so that we can interpret the results of the analyses from the standpoint of a de facto intervention study, although this study is indeed based on observational data. For a more detailed discussion on RDD, see Lee [31], Imbens and Lemieux [32], Lee and Lemieux [25], Ding et al. [8], and Takahashi [30].

As reported by Ding et al., using the Imbens and Kalyanaraman (IK) bandwidth, they revealed an optimal bandwidth of 26 days around a cutoff point (13 days before and 13 days after the cutoff point) [8]. Accordingly, we also used the IK bandwidth and found an optimal bandwidth of approximately 30 days around the cutoff point (15 days before and 15 days after the start of the emergency declarations). We envisaged a weekly seasonal effect of seven days; therefore, we used a bandwidth of 28 days around the cutoff point (14 days before and 14 days after the start of the emergency declaration). Just as in Ding et al. [8], as a standard sensitivity check for the choice of bandwidths [32, 33], we also used half the bandwidth (14 days around the cutoff point, 7 days before and 7 days after) and doubled the bandwidth (56 days around the cutoff point, 28 days before and 28 days after). These RDD models were used for all imputed datasets according to the missing rate.

All the RDD models were adjusted for age, sex, and educational levels as in Eq. (1), where $y$ is physical activity, ${x}_{1}$ is the date from February 1, 2020 (ranging from 1 to 547), $t$ is a binary indicator (0 = no emergency, 1 = emergency), $t{x}_{1}$ is the interaction term between ${x}_{1}$ and $t$, ${x}_{2}$ is age, ${x}_{3}$ is sex, ${x}_{4}$ is education levels, and $\varepsilon$ is the error term. Note that ${x}_{1}$ is the forcing variable, also known as the running variable, which determines the cutoff point [32, 33]. For the interaction term, ${x}_{1}$ $\mathrm{is}$ centered around the cutoff point (${x}_{1}-c$), so that ${\gamma }_{1}$ has a natural interpretation as the local average treatment effect at the cutoff point [33, 34]. The null hypothesis is ${\gamma }_{1}=0$, and the alternative hypothesis is ${\gamma }_{1}\ne 0$. Equation (1) is estimated using local data within 14-, 28-, and 56-day bandwidths from the cutoff point.

1 $$y={\beta }_{0}+{\beta }_{1}{x}_{1}+{\gamma }_{1}t+{\gamma }_{2}t{x}_{1}+{\beta }_{2}{x}_{2}+{\beta }_{3}{x}_{3}+{\beta }_{4}{x}_{4}+\varepsilon$$

Also, to check the robustness of the results for main datasets, we used RDD analyses for sub-groups, divided by age (75 ≥ or 75 <), sex, and median daily steps, for the first 14 days (from February 1, 2020). The results show point estimates with 95% confidence intervals. R ver. 4.0.4 (Vienna, Austria) was used to process all the analyses.

## Results

Table 1 shows the characteristics of the participants. The “mean” and “SD or %” columns are based on the missing data, where missing rows are listwise deleted. The “missing rate” column shows the percentage of missing observations in each variable. The “mean*” and “SD or %*” columns are based on the multiply-imputed data (M = 100).

A total of 1,056 participants are included in the primary analysis of the dataset with less than 50% of the maximum missing rate. The mean age of the participants is 74.5 with a SD of 5.9 years, and 597 (56.5%) are women. Age and sex are fully observed. The mean education level is 11.5 years with a SD of 2.4 years. The missing rate for education is only 0.3%; thus, the mean and the SD are approximately equal in the missing data and the multiply-imputed data. The overall missing rate for steps, LPA, and MVPA is 16.1% (mean number of valid measurement days: 458.9 ± 79.8 days and mean wearing time was 851.2 ± 154.4 min per day of valid measurement day); thus, the mean and the SD are no longer equal in the missing data and the multiply-imputed data. Our RDD analyses are based on the multiply-imputed data.

For the first 14 days (from February 1, 2020) of the observational period, mean steps are 6,698.1 with an SD of 4,043.6 steps (men: 7,596.2 ± 4,467.6, women: 6,007.6 ± 3,532.7), mean LPA time is 43.2 with an SD of 21.6 min per day (men: 43.6 ± 23.6, women: 42.9 ± 20.0), and mean MVPA is 34.5 with an SD of 27.1 min per day (men: 40.4 ± 30.7, women: 30.0 ± 23.1).

**Fig. 3:**
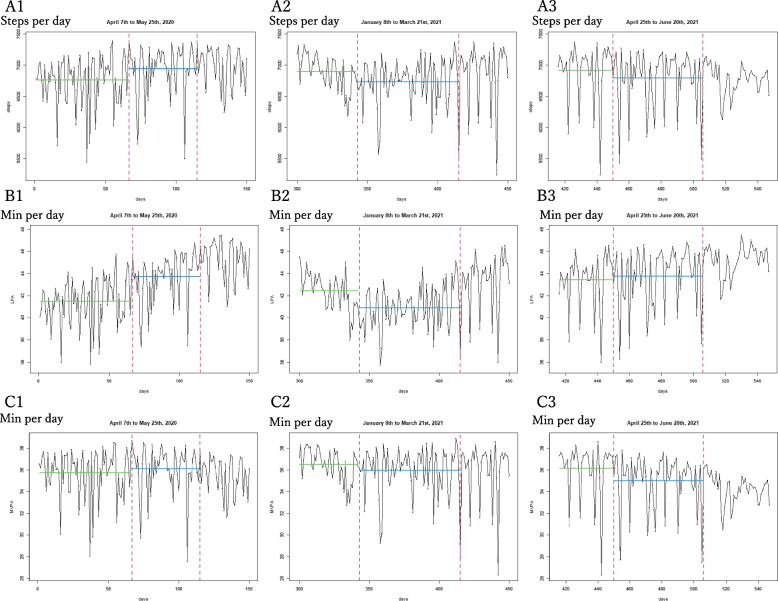
Mean daily physical activity of participants for the three emergency declaration periods. A1-3, daily steps; B1-3, daily time engaged in light intensity physical activity (LPA); C1-3, daily time engaged in moderate-to-vigorous intensity physical activity (MVPA). Green solid line for mean value before the emergency declaration, blue sold line for mean value during the emergency declaration period

Figure 2 displays the mean daily steps, mean daily LPA, and mean daily MVPA for the entire 547 days. The red, green, and blue vertical dashed lines represent the first, second, and third emergency declarations, respectively. Furthermore, in Fig. 3, the green and blue horizontal solid lines show the overall mean before and during the emergency declarations, respectively. For the first emergency declaration, we observed higher mean values of all physical activities (steps, LPA, and MVPA) after the declaration compared with before (Fig. 3: A1, B1, and C1). By contrast, for the second and third emergency declarations, we observed lower mean values of all physical activities (steps, LPA, and MVPA) after the declaration compared with before (Fig. 3: A2, A3, B2, C2, and C3, except B3). Overall, seasonal fluctuations are shown in Figs. 2 and 3. Therefore, it is hard to determine whether the emergency declarations had any effects on the mean daily steps, mean daily LPA, and mean daily MVPA. Thus, the RDD was required.

Table 2 presents the results of the sharp RDD for each physical activity. About the steps, with the bandwidth of 14 days, we observed statistically significant increase at the 5% error level for the first emergency declaration (coefficients[${\widehat{\gamma }}_{1}$]$,$ 964.3 steps, standard error [SE], 141.1, 95% confidence interval [CI], 687.8 to 1240.8), and statistically significant decrease for the second emergency declaration (${\widehat{\gamma }}_{1},$-609.7 steps, SE, 138.1, 95% CI, -880.3to -339.1), whereas there is no evidence for change in steps after the third declaration (${\widehat{\gamma }}_{1},$-259.2 steps, SE, 139.3, 95% CI, -532.2 to 13.8). With the bandwidth of 28 days, we observed statistically significant increase for the first declaration (${\widehat{\gamma }}_{1},$ 415.5 steps, SE, 98.3, 95% CI, 222.8 to 608.1), and statistically significant decrease for the third declaration (${\widehat{\gamma }}_{1},$-529.0 steps, SE, 96.0, 95% CI, -717.2 to -340.7), whereas there is no evidence for change in steps after the second declaration (${\widehat{\gamma }}_{1},$-117.0 steps, SE, 96.3, 95% CI, -305.8 to 71.8). With the bandwidth of 56 days, we observed statistically significant increase for the second declaration (${\widehat{\gamma }}_{1},$ 143.8 steps, SE, 67.7, 95% CI, 11.0 to 276.6), whereas there is no evidence for change in steps after the first declaration (${\widehat{\gamma }}_{1},$ -32.6 steps, SE, 69.3, 95% CI, -168.3 to 103.2) and the third declaration (${\widehat{\gamma }}_{1},$-108.4 steps, SE, 67.5, 95% CI, -240.7 to 23.9).

**Table 2:** Effects of the emergency on steps, LPA, and MVPA of participants (*n* = 1056) for Case 3

Outcomes	Emergency	Bandwidth	Coefficients (${\widehat{\gamma }}_{1}$)	SE	95% CI LL	95% CI UL	Observations (person-day)
Steps	1st April 7th, 2020	14 days	964.3	141.1	687.8	1240.8	14,784
		28 days	415.5	98.3	222.8	608.1	29,568
		56 days	-32.6	69.3	-168.3	103.2	59,136
	2nd January 8th, 2021	14 days	-609.7	138.1	-880.3	-339.1	14,784
		28 days	-117.0	96.3	-305.8	71.8	29,568
		56 days	143.8	67.7	11.0	276.6	59,136
	3rd April 25th, 2021	14 days	-259.2	139.3	-532.2	13.8	14,784
		28 days	-529.0	96.0	-717.2	-340.7	29,568
		56 days	-108.4	67.5	-240.7	23.9	59,136
LPA	1st April 7th, 2020	14 days	5.5	0.8	4.0	7.0	14,784
		28 days	2.4	0.5	1.4	3.5	29,568
		56 days	-0.3	0.4	-1.1	0.4	59,136
	2nd January 8th, 2021	14 days	-4.6	0.7	-6.0	-3.2	14,784
		28 days	-0.9	0.5	-1.8	0.1	29,568
		56 days	-0.4	0.4	-1.1	0.3	59,136
	3rd April 25th, 2021	14 days	-2.1	0.8	-3.7	-0.6	14,784
		28 days	-3.0	0.5	-4.0	-1.9	29,568
		56 days	-0.8	0.4	-1.5	-0.1	59,136
MVPA	1st April 7th, 2020	14 days	4.9	1.0	3.0	6.8	14,784
		28 days	2.2	0.7	0.9	3.5	29,568
		56 days	-0.1	0.5	-1.0	0.9	59,136
	2nd January 8th, 2021	14 days	-2.8	0.9	-4.6	-0.9	14,784
		28 days	-0.4	0.7	-1.7	0.9	29,568
		56 days	1.3	0.5	0.3	2.2	59,136
	3rd April 25th, 2021	14 days	-1.0	0.9	-2.8	0.9	14,784
		28 days	-2.6	0.6	-3.9	-1.4	29,568
		56 days	-0.4	0.5	-1.3	0.5	59,136

*SE* Standard error, *CI* Confidence interval, *LL* Lower limit, *UL* Upper limit, *LPA* Light intensity physical activity, *MVPA* Moderate-to-vigorous intensity physical activity

Models were adjusted for age, sex, and education

All models used cluster-robust standard errors

Multiply-imputed data (M = 100)

Observations were measured on a person-day basis

Days of half of the bandwidth duration before cut-off day are reference period

About the LPA, with the bandwidth of 14 days, we observed statistically significant increase for the first declaration (${\widehat{\gamma }}_{1},$ 5.5 min, SE, 0.8, 95% CI, 4.0 to 7.0), and statistically significant decrease for the second declaration (${\widehat{\gamma }}_{1},$-4.6 min, SE, 0.7, 95% CI, -6.3 to -3.2) and the third declaration (${\widehat{\gamma }}_{1},$-2.1 min, SE, 0.8, 95% CI, -3.7 to -0.6). With the bandwidth of 28 days, we observed statistically significant increase for the first declaration (${\widehat{\gamma }}_{1},$ 2.4 min, SE, 0.5, 95% CI, 1.4 to 3.5), and statistically significant decrease for the third declaration (${\widehat{\gamma }}_{1},$-3.0, minutes, SE, 0.5, 95% CI, -4.0 to -1.9), whereas there is no evidence for change in LPA after the second declaration (${\widehat{\gamma }}_{1},$-0.9 min, SE, 0.5, 95% CI, -1.8 to 0.1). With the bandwidth of 56 days, we observed statistically significant decrease for the third declaration (${\widehat{\gamma }}_{1},$-0.8 min, SE, 0.4, 95% CI, -1.5 to -0.1), whereas there is no evidence for change in LPA after the first declaration (${\widehat{\gamma }}_{1},$-0.3 min, SE, 0.4, 95% CI, -1.1 to 0.4) and the second declaration (${\widehat{\gamma }}_{1},$ -0.4 min, SE, 0.4, 95% CI, -1.1 to 0.3).

About the MVPA, with the bandwidth of 14 days, we observed statistically significant increase for the first declaration (${\widehat{\gamma }}_{1},$ 4.9 min, SE, 1.0, 95% CI, 3.0 to 6.8), and statistically significant decrease for the second declaration (${\widehat{\gamma }}_{1},$-2.8 min, SE, 0.9, 95% CI, -4.6 to -0.9), whereas there is no evidence for change in MVPA for the third declaration (${\widehat{\gamma }}_{1},$-1.0 min, SE, 0.9, 95% CI, -2.8 to 0.9). With the bandwidth of 28 days, we observed statistically significant increase for the first declaration (${\widehat{\gamma }}_{1},$ 2.2 min, SE, 0.7, 95% CI, 0.9 to 3.5), and statistically significant decrease for the third declaration (${\widehat{\gamma }}_{1},$-2.6, minutes, SE, 0.6, 95% CI, -3.9 to -1.4), whereas there is no evidence for change in LPA after the second declaration (${\widehat{\gamma }}_{1},$-0.4 min, SE, 0.7, 95% CI, -1.7 to 0.9). With the bandwidth of 56 days, we observed statistically significant increase for the second declaration (${\widehat{\gamma }}_{1},$ 1.3 min, SE, 0.5, 95% CI, 0.3 to 2.2), whereas there is no evidence for change in MVPA after the first declaration (${\widehat{\gamma }}_{1},$ -0.1 min, SE, 0.5, 95% CI, -1.0 to 0.9) and the third declaration (${\widehat{\gamma }}_{1},$-0.4 min, SE, 0.5, 95% CI, -1.3 to 0.5).

**Fig. 4:**
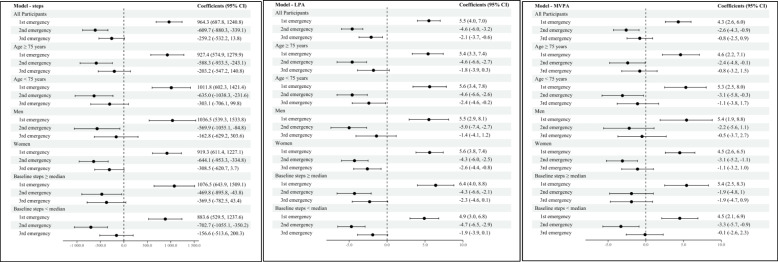
Beta coefficients and 95% CI of 14-day bandwidth for all participants and sub-groups. **A** steps; **B** light intensity physical activity (LPA); **C** moderate-to-vigorous intensity physical activity (MVPA). All models were adjusted for age, sex, and education. Multiply-imputed data (M = 100)

Figure 4 graphically presents the ${\widehat{\gamma }}_{1}$ and 95% CI from the RDD analyses, with forest plot, using the 14-day bandwidth. In the sub-groups, the results are consistent with the results for all the participants; all physical activity parameter estimates showed significant increase after the first emergency declaration, whereas significant decrease or no evidence of change for the second and third emergency declarations.

Supplemental Tables 1–3 show the participant characteristics, and Supplemental tables 4–6 display the results of the sharp RDD for the maximum missing rates of less than 75%, 25%, and 99.82%, respectively. The results from the datasets with the maximum missing rate of 99.82% shows consistent physical activity decrease for the first and third emergency declarations. There were no consistent change in physical activity for the maximum missing rate of less than 75% and 25% datasets, similar to the primary analysis. All physical activity parameter estimates were increased after the first declaration with the bandwidths of 14 and 28 days. Inconsistent effects through all the physical activity parameter estimates were observed for the second and third declarations.

## Discussion

In the current study, we tested the effect of the emergency declarations on physical activity including daily steps, daily time engaged in LPA and MVPA for community-dwelling older adults who had participated in the physical activity measurement project, using 547-day longitudinal cohort data. We hypothesized that the emergency declarations would have an adverse impact on the participants of the current study. Opposite to our hypothesis, negative impact was not consistently observed after the three times of emergency declarations. After the first declaration, all physical activity parameter estimates were increased, and the effects tended to be weakened and become less significant with the bandwidth widened. For the second declaration, results changed with the different bandwidths, especially for steps and MVPA. For the third declaration, physical activity tended to decrease but statistical significance was different among each physical activity parameter estimate. Even in the sub-group analyses by age, sex, and baseline steps, the effects of the emergency declarations with the 14-day bandwidth were similar to those for all participants. Inconsistent results were also observed for the other datasets generated by different criteria of missing rates. Besides, seasonal fluctuations were observed for all physical activities. Therefore, there was no evidence of significant adverse impact of the emergency declarations on physical activity, and seasonal changes might have affected the community-dwelling older adults with regularly monitoring their physical activity.

Previously, some reports negatively associated physical activity and the emergency declarations in Japan. For example, M. Yamada et al. measured total physical activity time via a web-based questionnaire survey after the emergency declarations and reported that total physical activity time decreased by 26.5% after the pandemic for community-dwelling older adults [20]. Also, Hisamatsu et al. showed that steps decreased after the emergency declarations in Japan [35]. These two reports which involved a broader participant seemingly contradict our findings according to the difference in characteristics of the participants.

Another previous study showed that city-lockdown did not have significant negative impact on daily steps for those people who were registered members of a smartphone-based physical activity-promoting program in Australia [36]. Participants of this previous study in Australia voluntarily enrolled, indicating a higher motivation to promote their physical activity levels and to self-monitor their progress [36]. Self-monitoring of physical activity increases self-monitoring by itself [37]. The participants in the current study had also been motivated to promote physical activity and had received regular physical activity measurement progress and feedback. These could have helped to maintain daily physical activity and could have weakened the adverse effects of the emergency declarations. In other words, our findings are in line with those in the previous study [36]. Furthermore, the participants in the current study regularly measured physical activity at the community facilities during the COVID-19 pandemic; in other words, participants could go out regularly. This could help to maintain physical activity. Although the COVID-19 pandemic and restrictions on daily activity by the official government may have a negative impact, the participants in the current study were estimated not to be strongly affected by the emergency declarations because of such conditions to maintain physical activity.

Furthermore, Ding et al. reported a dramatically decreased physical activity after the mandatory city-lock down in China, indicating a forced restraint physical activity level whereas people had high motivation or not [8]. In contrast, the current study showed no significant decline after the voluntary emergency declarations in Japan. Not only participant's characteristics, but also this difference in the implementation of infection control could affect the results. The voluntary emergency declaration in Japan did not have a significant impact on physical activity decline among community-dwelling older adults who regularly measure their physical activity, although it is estimated to decrease the COVID-19 infection rate [10].

The major strengths of the current study included: (1) the large sample size; (2) the objective measurement of physical activity data via tri-axial accelerometers; (3) the use of consecutive longitudinal data recorded over a long period; and (4) a quasi-experiment using the RDD for causal analysis. This study examined the effect of the emergency declarations on detailed physical activity data, including quantity (i.e., steps) and quality (i.e., LPA and MVPA).

This study has some potential limitations. First, generalizability is limited due to the characteristics of the participants in the current study, who might have preserved physical activity and relatively better health conditions. Self-monitoring increases physical activity [37]. Besides, non-participation in accelerometry was linked to several cardiometabolic risk variables in a previous study [38]. Second, the accelerometer does not measure several types of physical activity measured by stationary machines and bike exercises. We might have underestimated the effect for the participants who underwent those exercises regularly because several training and sport facilities had closed during the emergency declarations. Third, we did not identify the consistency of the results using the sub-analysis for other factors which affect physical activity such as physical function, socioeconomic and environmental factors, cognitive function, depressive symptom, and chronic disease, because the measurement period of the current study was more than three years after the baseline survey. Fourth, the main area of the study was limited to Takahama City, a regional city with a lower population density than major cities in Japan, such as Tokyo, Osaka, and Nagoya. Because the frequency of contact with other people was less in Takahama City than in those major cities, the participants in Takahama City may have had more opportunities to go out. Fifth, participants had measured daily physical activity since the baseline survey of NCGG-SGS conducted between 2015 and 2017. We asked all the participants of NCGG-SGS to wear accelerometers longitudinally, but some participants dropped out. Those participants were not included in the analysis. Participants who did not drop out might have had better physical function and health conditions, allowing them to maintain physical activity during the pandemic. Mean values of time spent in MVPA were higher, compared with those in a previous study that used the accelerometer with a similar algorithm [39]. Therefore, the effect of the emergency declarations on physical activity for the participants in the current study was affected due to such participant's characteristics and environmental aspects.

## Conclusions

In conclusion, significant negative impact of the voluntary emergency declarations by the COVID-19 in Japan on physical activity was not observed among the community-dwelling older adults who regularly monitored their physical activity. As noted above, generalizability in the current study is limited due to the characteristics of the participants, by which we implied that self-monitoring would increase physical activity. This may further suggest an implication for practice in terms of public health as follows: Regular self-monitoring may have the potential to mitigate the negative effect of emergency declarations on the physical activity level for older adults, assuming that these older adults share similar characteristics with the participants of the current study.

## Supplementary Information

**Additional file 1: Supplemental table 1.** Participants’ characteristics for Case 1.** Supplemental table 2.** Participants’ characteristics for Case 2.** Supplemental table 3.** Participants’ characteristics for Case 4.** Supplemental table 4.** Effects of the emergency on steps, LPA, and MVPA for Case 1.** Supplemental table 5.** Effects of the emergency on steps, LPA, and MVPA for Case 2.** Supplemental table 6.** Effects of the emergency on steps, LPA, and MVPA for Case 4.

## Data Availability

To request the data from this study, please contact the co-first author. Ippei Chiba. Department of Preventive Gerontology, Centre for Gerontology and Social Science, National Centre for Geriatrics and Gerontology. 7–430, Morioka-cho, Obu, City, Aichi 474–8511, Japan. E-mail: ichibapt@hotmail.com. To request the R-code to replicate the results in the current, please contact the other co-first author. Masayoshi Takahashi. School of Information and Data Sciences, Nagasaki University, Nagasaki, 852–8521, Japan. E-mail: m-takahashi@nagasaki-u.ac.jp.

## Abbreviations

LPA: Light physical activity
MVPA: Moderate-to-vigorous physical activity
RDD: Regression discontinuity design
