# Supplementary material for: Association between COVID-19 emergency declarations and physical activity among community-dwelling older adults enrolled in a physical activity measurement program: Evidence from a retrospective observational study using the regression discontinuity design
Source: BMC Public Health. 2023 May 30;23:998. doi: 10.1186/s12889-023-15932-0 (PMC10226879; doi:10.1186/s12889-023-15932-0)
Supplement: Supplementary file 1 — Additional file 1: Supplemental table 1. Participants’ characteristics for Case 1. Supplemental table 2. Participants’ characteristics for Case 2. Supplemental table 3. Participants’ characteristics for Case 4. Supplemental table 4. Effects of the emergency on steps, LPA, and MVPA for Case 1. Supplemental table 5. Effects of the emergency on steps, LPA, and MVPA for Case 2. Supplemental table 6. Effects of the emergency on steps, LPA, and MVPA for Case 4. [file 12889_2023_15932_MOESM1_ESM.docx]

Supplemental File

The baseline survey originally included 4,092 participants. We recorded physical activity from February 2020 to July 2021, which is 547 days. Therefore, the ideal sample size is 4,092*547 = 2,238,324 person-days.

However, only 1,773 participants had recorded at least one day of physical activity, which makes our sample size 1,773*547 = 969,831 person-days, after multiple imputation. This was the original dataset that the first manuscript used (let us call this Case 1), because 2,319 participants had no data on physical activity, which makes it impossible to use multiple imputation to take care of the missing values of physical activity. When there are zero observations, it is impossible to create an imputation model.

Among these 1,773 participants, there were 30 participants whose physical activity was recorded only one day in 547 days. For these participants, it is still possible to use multiple imputation to take care of the missing values, but 546 observations are simulated values. This means that the maximum missing rate at the person level is 99.82%. As the reviewer was concerned, this may pose a serious problem in inference. There are no ideal solutions to this problem, and we must face trade-offs. Therefore, we decided to conduct analyses based on the following three patterns of data.

(Case 2) 137 days or more: 1,289 participants

There are 1,289 participants whose physical activity was recorded at least 137 days in 547 days. This means that the maximum missing rate at the person level is about 75%. Our sample size after multiple imputation is 1,289*547 = 705,083 person-days.

(Case 3) 274 days or more: 1,056 participants

There are 1,056 participants whose physical activity was recorded at least 274 days in 547 days. This means that the maximum missing rate at the person level is about 50%. Our sample size after multiple imputation is 1,056*547 = 577,632 person-days.

(Case 4) 411 days or more: 768 participants

There are 768 participants whose physical activity was recorded at least 411 days in 547 days. This means that the maximum missing rate at the person level is about 25%. Our sample size after multiple imputation is 768*547 = 420,096 person-days.

We decided to choose Case 3 (50% missing rate) as our main analysis to be reported in the main body of the manuscript, because several short-term measurement studies used 50% or more adherence data, despite non-imputed data. Cases 1, 2, and 4 are reported in this supplemental file.

| **Supplemental table 1**　Participants’ characteristics for Case 1 | | | | | |
| --- | --- | --- | --- | --- | --- |
| Variables | mean | SD or % | missing rate | mean* | SD or %* |
| Age, years | 74.6 | (6.3) | 0.0 |  |  |
| Age ≥ 75 years, n (%) | 80.1 | (3.9) | 0.0 |  |  |
| Women, n (%) | 956 | (53.9) | 0.0 |  |  |
| Education, years | 11.4 | (2.3) | 7.1 | 11.4 | (2.3) |
| Step counts at first 14 days, steps/day |  |  |  |  |  |
| All participants | 6446.9 | (4156.7) | 36.0 | 6781.5 | (3948.2) |
| Men | 7324.9 | (4644.8) | 38.3 | 7602.4 | (4264.7) |
| Women | 5732.3 | (3524.2) | 34.0 | 6080.0 | (3507.1) |
| LPA time at first 14 days, min/day |  |  |  |  |  |
| All participants | 40.4 | (21.9) | 36.0 | 41.0 | (21.3) |
| Men | 42.7 | (24.5) | 38.3 | 41.7 | (23.5) |
| Women | 41.9 | (19.9) | 34.0 | 40.5 | (19.3) |
| MVPA time at first 14 days, min/day |  |  |  |  |  |
| All participants | 33.8 | (27.4) | 36.0 | 36.2 | (26.1) |
| Men | 38.6 | (31.7) | 38.3 | 41.3 | (28.6) |
| Women | 28.3 | (22.8) | 34.0 | 31.8 | (22.9) |
| SD, standard deviation; LPA, light intensity physical activity; MVPA, moderate-to-vigorous intensity physical activity. | | | | | |
| Education data was collected during the baseline survey conducted from 2015 to 2017. Age information was collected as of February 1st, 2022, while physical activity data were gathered between February 1st and 14th of the same year. | | | | | |
| Mean number of valid measurement days: 458.9 ± 79.8 days, and mean wearing time was 851.2 with standard deviation of 154.4 minutes per day of valid measurement day | | | | | |
| *Data for "Women, yes" and "Aged 75 years or above" are presented as n (%). | | | | | |
| *Multiply-imputed data (M=100). |  |  |  |  |  |

| **Supplemental table 2**　Participants’ characteristics for Case 2 | | | | | |
| --- | --- | --- | --- | --- | --- |
| Variables | mean | SD or % | missing rate | mean* | SD or %* |
| Age, years | 74.5 | (6.0) | 0.0 |  |  |
| Age ≥ 75 years, n (%) | 621 | (48.2) | 0.0 |  |  |
| Women, n (%) | 707 | (54.8) | 0.0 |  |  |
| Education, years | 11.5 | (2.4) | 0.3 | 11.5 | (2.4) |
| Step counts at first 14 days, steps/day |  |  |  |  |  |
| All participants | 6479.5 | (4130.0) | 24.5 | 6717.1 | (3997.4) |
| Men | 7382.6 | (4645.5) | 27.0 | 7554.2 | (4381.7) |
| Women | 5779.2 | (3525.0) | 22.5 | 6027.9 | (3503.8) |
| LPA time at first 14 days, min/day |  |  |  |  |  |
| All participants | 42.4 | (22.0) | 24.5 | 43.0 | (21.6) |
| Men | 42.8 | (24.4) | 27.0 | 43.4 | (23.4) |
| Women | 42.1 | (19.9) | 22.5 | 42.8 | (20.1) |
| MVPA time at first 14 days, min/day |  |  |  |  |  |
| All participants | 33.2 | (27.7) | 24.5 | 34.7 | (26.8) |
| Men | 39.1 | (31.9) | 27.0 | 40.2 | (30) |
| Women | 28.6 | (22.9) | 22.5 | 30.2 | (22.9) |
| SD, standard deviation; LPA, light intensity physical activity; MVPA, moderate-to-vigorous intensity physical activity. | | | | | |
| Education data was collected during the baseline survey conducted from 2015 to 2017. Age information was collected as of February 1st, 2022, while physical activity data were gathered between February 1st and 14th of the same year. | | | | | |
| Mean number of valid measurement days: 413.0 ± 122.8 days, and mean wearing time was 843.1 with standard deviation of 154.9 minutes per day of valid measurement day | | | | | |
| *Data for "Women, yes" and "Aged 75 years or above" are presented as n (%). | | | | | |
| *Multiply-imputed data (M=100). |  |  |  |  |  |

| **Supplemental table 3**　Participants’ characteristics for Case 4 | | | | | |
| --- | --- | --- | --- | --- | --- |
| Variables | mean | SD or % | missing rate | mean* | SD or %* |
| Age, years | 74.5 | (5.8) | 0.0 |  |  |
| Age ≥ 75 years, n (%) | 362 | (47.1) | 0.0 |  |  |
| Women, n (%) | 597 | (56.5) | 0.0 |  |  |
| Education, years | 11.6 | (2.4) | 0.1 | 11.6 | (2.4) |
| Step counts at first 14 days, steps/day |  |  |  |  |  |
| All participants | 6616.7 | (4075.7) | 8.4 | 6687.9 | (4032.6) |
| Men | 7518.2 | (4500.7) | 9.0 | 7569.5 | (4423.1) |
| Women | 5973.9 | (3608.2) | 7.9 | 6051.4 | (3593.2) |
| LPA time at first 14 days, min/day |  |  |  |  |  |
| All participants | 43.4 | (21.7) | 8.4 | 43.6 | (21.6) |
| Men | 43.4 | (23.7) | 9.0 | 43.6 | (23.4) |
| Women | 43.4 | (20.1) | 7.9 | 43.6 | (20.1) |
| MVPA time at first 14 days, min/day |  |  |  |  |  |
| All participants | 33.8 | (27.4) | 8.4 | 34.3 | (27.1) |
| Men | 39.8 | (31.0) | 9.0 | 40.1 | (30.4) |
| Women | 29.6 | (23.5) | 7.9 | 30.1 | (23.5) |
| SD, standard deviation; LPA, light intensity physical activity; MVPA, moderate-to-vigorous intensity physical activity. | | | | | |
| Education data was collected during the baseline survey conducted from 2015 to 2017. Age information was collected as of February 1st, 2022, while physical activity data were gathered between February 1st and 14th of the same year. | | | | | |
| Mean number of valid measurement days: 501.3 ± 39.2 days, and mean wearing time was 869.1 with standard deviation of 155.4 minutes per day of valid measurement day | | | | | |
| *Data for "Women, yes" and "Aged 75 years or above" are presented as n (%). | | | | | |
| *Multiply-imputed data (M=100). |  |  |  |  |  |

| **Supplemental table 4**　Effects of the emergency on steps, LPA, and MVPA for Case 1 | | | | | | | | | |
| --- | --- | --- | --- | --- | --- | --- | --- | --- | --- |
| Outcomes | Emergency | Bandwidth | Coefficients ($\hat{\gamma}_{1}$) | SE | | 95% CI LL | | 95% CI UL | Observations  (person-day) |
| Steps | 1st  April 7th, 2020 | 14 days | -326.041 | 28.500 | | -381.902 | | -270.179 | 24822 |
|  |  | 28 days | -52.762 | 9.709 | | -33.731 | | -71.793 | 49644 |
|  |  | 56 days | -3.178 | 3.567 | | -10.170 | | 3.814 | 99288 |
|  | 2nd  January 8th, 2021 | 14 days | -50.125 | 27.855 | | -104.722 | | 4.472 | 24822 |
|  |  | 28 days | 15.722 | 9.645 | | -3.184 | | 34.628 | 49644 |
|  |  | 56 days | 8.686 | 3.395 | | 2.031 | | 15.340 | 99288 |
|  | 3rd  April 25th, 2021 | 14 days | -116.106 | 27.302 | | -169.619 | | -62.592 | 24822 |
|  |  | 28 days | -49.442 | 9.579 | | -68.217 | | -30.667 | 49644 |
|  |  | 56 days | -14.461 | 3.409 | | -21.143 | | -7.778 | 99288 |
| LPA | 1st  April 7th, 2020 | 14 days | -1.619 | 0.152 | | -1.917 | | -1.320 | 24822 |
|  |  | 28 days | -0.267 | 0.055 | | -0.374 | | -0.160 | 49644 |
|  |  | 56 days | 0.016 | 0.020 | | -0.023 | | 0.055 | 99288 |
|  | 2nd  January 8th, 2021 | 14 days | -0.178 | 0.158 | | -0.488 | | 0.131 | 24822 |
|  |  | 28 days | 0.301 | 0.053 | | 0.405 | | 0.197 | 49644 |
|  |  | 56 days | 0.082 | 0.019 | | 0.044 | | 0.119 | 99288 |
|  | 3rd  April 25th, 2021 | 14 days | -0.716 | 0.160 | | -1.029 | | -0.403 | 24822 |
|  |  | 28 days | -0.312 | 0.059 | | -0.427 | | -0.196 | 49644 |
|  |  | 56 days | -0.064 | 0.020 | | -0.103 | | -0.024 | 99288 |
| MVPA | 1st  April 7th, 2020 | 14 days | -1.809 | 0.191 | | -2.184 | | -1.435 | 24822 |
|  |  | 28 days | -0.286 | 0.066 | | -0.156 | | -0.416 | 49644 |
|  |  | 56 days | -0.024 | 0.024 | | 0.023 | | -0.070 | 99288 |
|  | 2nd  January 8th, 2021 | 14 days | -0.309 | 0.188 | | -0.677 | | 0.059 | 24822 |
|  |  | 28 days | -0.004 | 0.066 | | -0.133 | | 0.124 | 49644 |
|  |  | 56 days | 0.028 | 0.023 | | -0.017 | | 0.073 | 99288 |
|  | 3rd  April 25th, 2021 | 14 days | -0.554 | 0.186 | | -0.918 | | -0.190 | 24822 |
|  |  | 28 days | -0.242 | 0.064 | | -0.368 | | -0.116 | 49644 |
|  |  | 56 days | -0.088 | 0.023 | | -0.133 | | -0.043 | 99288 |
| Multiply-imputed data (M=100). | | |  |  | |  | |  |  |
| Observations were measured on a person-day basis. | | | |  | |  | |  |  |
| Days of half of the bandwidth duration before cut-off day are reference period. | | | | | | | | | |
| SE, standard error; CI, confidence interval; LL, lower limit; UL, upper limit; LPA, light intensity physical activity; MVPA, moderate-to-vigorous intensity physical activity. | | | | | | | | | |
| Models were adjusted for age, sex, and education. | | | | |  | |  |  |  |
| All models used cluster-robust standard errors. | | | | |  | |  |  |  |

| **Supplemental table 5**　Effects of the emergency on steps, LPA, and MVPA for Case 2 | | | | | | | | | |
| --- | --- | --- | --- | --- | --- | --- | --- | --- | --- |
| Outcomes | Emergency | Bandwidth | Coefficients ($\hat{\gamma}_{1}$) | SE | | 95% CI LL | | 95% CI UL | Observations  (person-day) |
| Steps | 1st  April 7th, 2020 | 14 days | 839.2 | 128.7 | | 586.9 | | 1091.5 | 18046 |
|  |  | 28 days | 366.3 | 88.9 | | 192.0 | | 540.6 | 36092 |
|  |  | 56 days | -37.7 | 62.2 | | -159.7 | | 84.2 | 72184 |
|  | 2nd  January 8th, 2021 | 14 days | -574.2 | 128.1 | | -825.3 | | -323.1 | 18046 |
|  |  | 28 days | -141.3 | 87.2 | | -312.3 | | 29.7 | 36092 |
|  |  | 56 days | 100.3 | 62.3 | | -21.8 | | 222.4 | 72184 |
|  | 3rd  April 25th, 2021 | 14 days | -224.5 | 125.3 | | -470.2 | | 21.1 | 18046 |
|  |  | 28 days | -448.1 | 88.3 | | -621.2 | | -275 | 36092 |
|  |  | 56 days | -87.6 | 62.2 | | -209.6 | | 34.3 | 72184 |
| LPA | 1st  April 7th, 2020 | 14 days | 4.8 | 0.7 | | 3.4 | | 6.2 | 18046 |
|  |  | 28 days | 2.2 | 0.5 | | 1.2 | | 3.2 | 36092 |
|  |  | 56 days | -0.3 | 0.3 | | -1.0 | | 0.4 | 72184 |
|  | 2nd  January 8th, 2021 | 14 days | -4.1 | 0.7 | | -5.5 | | -2.7 | 18046 |
|  |  | 28 days | -0.8 | 0.5 | | -1.8 | | 0.1 | 36092 |
|  |  | 56 days | -0.4 | 0.3 | | -1.0 | | 0.3 | 72184 |
|  | 3rd  April 25th, 2021 | 14 days | -1.9 | 0.7 | | -3.3 | | -0.5 | 18046 |
|  |  | 28 days | -2.6 | 0.5 | | -3.6 | | -1.6 | 36092 |
|  |  | 56 days | -0.7 | 0.4 | | -1.4 | | 0.0 | 72184 |
| MVPA | 1st  April 7th, 2020 | 14 days | 4.3 | 0.9 | | 2.6 | | 6.0 | 18046 |
|  |  | 28 days | 1.9 | 0.6 | | 0.7 | | 3.1 | 36092 |
|  |  | 56 days | -0.1 | 0.4 | | -0.9 | | 0.7 | 72184 |
|  | 2nd  January 8th, 2021 | 14 days | -2.6 | 0.9 | | -4.3 | | -0.9 | 18046 |
|  |  | 28 days | -0.6 | 0.6 | | -1.8 | | 0.5 | 36092 |
|  |  | 56 days | 0.9 | 0.4 | | 0.1 | | 1.7 | 72184 |
|  | 3rd  April 25th, 2021 | 14 days | -0.8 | 0.9 | | -2.5 | | 0.9 | 18046 |
|  |  | 28 days | -2.2 | 0.6 | | -3.3 | | -1.0 | 36092 |
|  |  | 56 days | -0.3 | 0.4 | | -1.1 | | 0.5 | 72184 |
| Multiply-imputed data (M=100). | | |  |  | |  | |  |  |
| Observations were measured on a person-day basis. | | | |  | |  | |  |  |
| Days of half of the bandwidth duration before cut-off day are reference period. | | | | | | | | | |
| SE, standard error; CI, confidence interval; LL, lower limit; UL, upper limit; LPA, light intensity physical activity; MVPA, moderate-to-vigorous intensity physical activity. | | | | | | | | | |
| Models were adjusted for age, sex, and education. | | | | |  | |  |  |  |
| All models used cluster-robust standard errors. | | | | |  | |  |  |  |

| **Supplemental table 6**　Effects of the emergency on steps, LPA, and MVPA for Case 4 | | | | | | | | | |
| --- | --- | --- | --- | --- | --- | --- | --- | --- | --- |
| Outcomes | Emergency | Bandwidth | Coefficients ($\hat{\gamma}_{1}$) | SE | | 95% CI LL | | 95% CI UL | Observations  (person-day) |
| Steps | 1st  April 7th, 2020 | 14 days | 1060.4 | 157.7 | | 751.3 | | 1369.5 | 10752 |
|  |  | 28 days | 411.3 | 110.5 | | 194.6 | | 628.0 | 21504 |
|  |  | 56 days | -63.0 | 78.2 | | -216.2 | | 90.3 | 43008 |
|  | 2nd  January 8th, 2021 | 14 days | -637.8 | 158.7 | | -948.9 | | -326.7 | 10752 |
|  |  | 28 days | -113.0 | 110.7 | | -330.0 | | 104.0 | 21504 |
|  |  | 56 days | 160.9 | 78.5 | | 7.1 | | 314.8 | 43008 |
|  | 3rd  April 25th, 2021 | 14 days | -291.9 | 158.4 | | -602.4 | | 18.6 | 10752 |
|  |  | 28 days | -624.4 | 111.2 | | -842.3 | | -406.4 | 21504 |
|  |  | 56 days | -153.6 | 78.5 | | -307.5 | | 0.4 | 43008 |
| LPA | 1st  April 7th, 2020 | 14 days | 6.6 | 0.9 | | 4.9 | | 8.3 | 10752 |
|  |  | 28 days | 2.8 | 0.6 | | 1.6 | | 4.0 | 21504 |
|  |  | 56 days | -0.3 | 0.4 | | -1.1 | | 0.6 | 43008 |
|  | 2nd  January 8th, 2021 | 14 days | -5.0 | 0.8 | | -6.6 | | -3.4 | 10752 |
|  |  | 28 days | -0.9 | 0.6 | | -2.0 | | 0.2 | 21504 |
|  |  | 56 days | -0.4 | 0.4 | | -1.2 | | 0.4 | 43008 |
|  | 3rd  April 25th, 2021 | 14 days | -2.0 | 0.9 | | -3.8 | | -0.3 | 10752 |
|  |  | 28 days | -3.1 | 0.6 | | -4.3 | | -1.8 | 21504 |
|  |  | 56 days | -0.9 | 0.4 | | -1.7 | | 0.0 | 43008 |
| MVPA | 1st  April 7th, 2020 | 14 days | 5.2 | 1.1 | | 3.1 | | 7.3 | 10752 |
|  |  | 28 days | 2.0 | 0.7 | | 0.5 | | 3.5 | 21504 |
|  |  | 56 days | -0.3 | 0.5 | | -1.3 | | 0.7 | 43008 |
|  | 2nd  January 8th, 2021 | 14 days | -2.8 | 1.1 | | -4.9 | | -0.7 | 10752 |
|  |  | 28 days | -0.4 | 0.8 | | -1.9 | | 1.1 | 21504 |
|  |  | 56 days | 1.4 | 0.5 | | 0.4 | | 2.5 | 43008 |
|  | 3rd  April 25th, 2021 | 14 days | -1.3 | 1.1 | | -3.4 | | 0.8 | 10752 |
|  |  | 28 days | -3.4 | 0.7 | | -4.8 | | -1.9 | 21504 |
|  |  | 56 days | -0.8 | 0.5 | | -1.8 | | 0.2 | 43008 |
| Multiply-imputed data (M=100). | | |  |  | |  | |  |  |
| Observations were measured on a person-day basis. | | | |  | |  | |  |  |
| Days of half of the bandwidth duration before cut-off day are reference period. | | | | | | | | | |
| SE, standard error; CI, confidence interval; LL, lower limit; UL, upper limit; LPA, light intensity physical activity; MVPA, moderate-to-vigorous intensity physical activity. | | | | | | | | | |
| Models were adjusted for age, sex, and education. | | | | |  | |  |  |  |
| All models used cluster-robust standard errors. | | | | |  | |  |  |  |
